# Supplementary material for: Long-term exposure to outdoor air pollution and asthma in low-and middle-income countries: A systematic review protocol
Source: PLoS One. 2023 Jul 20;18(7):e0288667. doi: 10.1371/journal.pone.0288667 (PMC10358890; doi:10.1371/journal.pone.0288667)
Supplement: Supplementary file 3 [file pone.0288667.s003.docx]

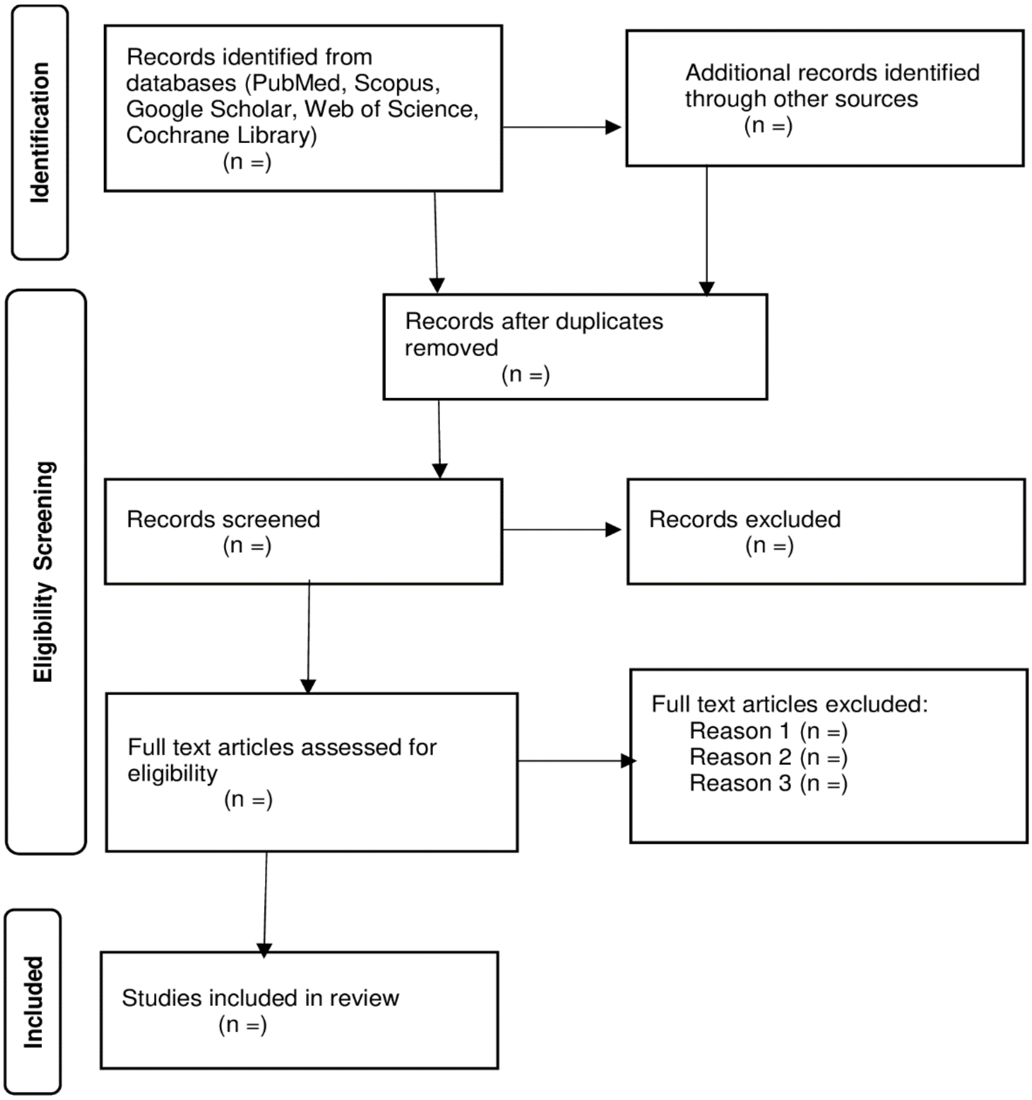

S1 Fig. PRISMA flowchart of the study identification and selection process
