# Supplementary material for: Long-term exposure to outdoor air pollution and asthma in low-and middle-income countries: A systematic review protocol
Source: PLoS One. 2023 Jul 20;18(7):e0288667. doi: 10.1371/journal.pone.0288667 (PMC10358890; doi:10.1371/journal.pone.0288667)
Supplement: Attachment — Submitted filename: Response to Reviewers_12may23.docx [file pone.0288667.s004.docx]

Dear PLOS ONE academic editor Dr Haruna Musa Moda and reviewers,

Thank you for giving us the opportunity to submit a revised draft of our manuscript titled “Long-term health effects of outdoor air pollution on asthma and respiratory symptoms: a systematic review and meta-analysis” to *PLOS ONE*. We appreciate the time and effort that you have dedicated to providing your valuable feedback on our manuscript. We have carefully revised the manuscript according to the reviewers’ insightful comments and have provided point-by-point responses as follows, and we have highlighted the changes within the manuscript. We greatly appreciate the Reviewers’ comments, they enabled us to improve the quality of our paper substantially.

**Journal Requirements:**

When submitting your revision, we need you to address these additional requirements.

1. Please ensure that your manuscript meets PLOS ONE's style requirements, including those for file naming. The PLOS ONE style templates can be found at <https://journals.plos.org/plosone/s/file?id=wjVg/PLOSOne_formatting_sample_main_body.pdf> and 
<https://journals.plos.org/plosone/s/file?id=ba62/PLOSOne_formatting_sample_title_authors_affiliations.pdf>

**Response:** We have adhered to the PLOS ONE’s style requirements in the revised version of the manuscript

2. We note that the original protocol file you uploaded contains a confidentiality notice indicating that the protocol may not be shared publicly or published. Please note, however, that the PLOS Editorial Policy requires that the original protocol be published alongside your manuscript in the event of acceptance. Please note that should your paper be accepted, all content including the protocol will be published under the Creative Commons Attribution (CC BY) 4.0 license, which means that it will be freely available online, and any third party is permitted to access, download, copy, distribute, and use these materials in any way, even commercially, with proper attribution.

**Response:** We have removed the confidentiality notice from the revised version of the manuscript and have added a statement that we will be able to publish this protocol under CC BY 4.0.

Therefore, we ask that you please seek permission from the study sponsor or body imposing the restriction on sharing this document to publish this protocol under CC BY 4.0 if your work is accepted. We kindly ask that you upload a formal statement signed by an institutional representative clarifying whether you will be able to comply with this policy. Additionally, please upload a clean copy of the protocol with the confidentiality notice (and any copyrighted institutional logos or signatures) removed.

3. We noticed you have some minor occurrences of overlapping text with the following previous publication(s), which needs to be addressed:

- <https://www.frontiersin.org/articles/10.3389/fped.2021.827507/full>

In your revision ensure you cite all your sources (including your own works), and quote or rephrase any duplicated text outside the methods section. A further consideration is dependent on these concerns being addressed.

**Response:** We have not seen the review article on global research on perinatal palliative care that you refer to before. Any similarity and overlapping text with this paper is therefore due to chance. We suspect there will be some degree of overlapping text between all systematic review papers within the medical field, as there are many formal requirements to this kind of paper needs to adhere to. Please let us know if we need to revise any sections of our text due to this coincidence, we will of course be happy to oblige.

**Comments from Reviewers**

**Reviewer #1:**

**Comment 1:** *Make sure to accentuate the findings of the systematic review that relates to /answers the review question you stated in your protocol.*

**Response:** Thank you for pointing this out, we will indeed accentuate the findings relating to the review question in our systematic review and keep this protocol paper vivid in mind when performing the systematic review.

**Reviewer #2:** Detailed Review
The current manuscript details a study protocol for carrying out a systematic review of long-term exposure to outdoor air pollution and its links to asthma prevalence in low-and middle-income countries (LMIC).

**General comment 1:** *The English language in the manuscript needs to be more polished
e.g., 1 - Refer to lines 61 to 63. The wordings are not right
e.g., 2 - Instead of using Dual et al., Orlenno et al., and so forth in the paper, rewrite these sentences to bring in more uniformity for the reader.*

**Response:** Thank you for pointing this out. We agree with this comment and have gone through the manuscript rephrasing wordings in better English where needed to improve the reader’s understanding.

**e.g., 1:** We have corrected the wording. The change can for example be found on page 3, paragraph 3, and lines 63-64.

**e.g., 2-** We have rewritten the sentences by incorporating the names of the authors using in-text Vancouver referencing style all through the manuscript (writing, for example, Duan and co-workers instead of Duan et al. on page 3, paragraph 2, and lines 60).

**General comment 2:** *Certain sections should be rewritten more concisely
E.g. – Section on “Assessment of certainty of evidence across studies”
Here, once the author expands the GRADE approach and cites appropriately, there is no need to write in detail and define it. One of the important purposes of citing/quoting references is to refer the reader to sources that can provide more information on the context being discussed and avoid detailing the concepts or procedures again.*

**Response:** Agree. We have, accordingly, revised this sentence and we have deleted the detailed description. We have made the changes on page 11, lines 235-237.

**Major comment 1:** *Lines 84-85 – Mentions systematic overview from LMICs is lacking. This definitely is an understatement as recent systematic reviews and meta-analyses are available on this topic (*[*https://doi.org/10.1016/j.envres.2022.114604*](https://doi.org/10.1016/j.envres.2022.114604)*;* [*https://doi.org/10.1016/j.atmosenv.2021.118422*](https://doi.org/10.1016/j.atmosenv.2021.118422)*).
Hence, the introduction needs to be rewritten with a better emphasis on the need for the current proposed review and how it is different/unique from other existing reviews on the topic.*

**Response:** We thank the reviewers for the two review papers’ recommendations. Our statement about how systematic overviews from LMICs are lacking refers to overviews on air pollution and asthma in adults. We agree this should be more clearly specified and have rewritten the Introduction accordingly. We have also included the two suggested references (page 4, lines 89-93).

**Major comment 2:** *Lines 249-251 - The expected outcomes of the review are overstated. How are the authors linking a review of this kind to directly inform policymaking?*

**Response:** we agree the expected outcomes may have been worded a bit too ambitiously, and we have revised the manuscript accordingly. Although systematic review papers such as our planned paper are indeed suitable to inform policymaking- through summarizing large amounts of information, identifying positive and negative effects of various exposures, and identifying gaps in medical research- we do not have a direct link with the policymakers in LMICs and can therefore not assume that our paper will directly inform policymaking. We have kept the potential for policymaking in the manuscript but have acknowledged that we cannot be certain that our paper will be used for this purpose (page 5, lines 93-98, page 12, lines 258-260, lines 262-264).

**Major comment 3:** *In their inclusion criteria, the authors mention “Long term” and “≥1 year”. Typically, at least a period covering more than one annual cycle or with repetitive annual seasonal cycles are referred to as long term. Hence, use a different jargon or increase the exposure duration to “≥2 years” if feasible.*
**Response:**  Although we agree that more than one annual cycle is ideal for looking at long-term exposures, the purpose of this planned systematic review is to gather an overview of all long-term air pollution exposure papers focusing on asthma in adults in LMICs. To not miss out on any papers, we have chosen the definition of long-term exposure from the 2021 WHO Global air quality guidelines. In these guidelines, long-term exposure is defined as “a mean of one or several years” while short-term exposure is “measures over minutes to days”. We have specified the reason for our definition of long-term exposure in the revised manuscript (page 6, lines 128-129).

**Minor comment 1:** *Lines 36 to 42 – The historical statements given here are of little importance to the context. The authors should rewrite these in a better way leading the reader to the topic.*

**Response:** Thank you for pointing this out. We have, accordingly, changed the first paragraph in the introduction section for better understanding. This can be found on pages 2-3, lines 38-46.

**Minor comment 2:** *Lines 125-126 – “Outcomes that include asthma, respiratory symptoms” – Such outcomes can also result from biological agents. So care should be taken while screening articles and this needs to be explicitly mentioned in the inclusion/exclusion criteria*

**Response:** Agree. Health outcomes that were identified in this systematic review are now explicitly mentioned in the inclusion/exclusion criteria. This can be found on page 6, lines 135-136.

**Minor comment 3:** *Introduce expanded forms of abbreviations before start using them (e.g., RoB)*

**Response:** We agree with this and have incorporated your suggestion throughout the manuscript. The expanded form of RoB can be found on page 11, and line 242.

**Minor comment 4:** *Please check for the appropriate use of subscripts and postscripts throughout the manuscript including the reference section.*

**Response:** We agree with this and have incorporated your suggestion throughout the manuscript. These changes can for example be found on page 13, lines 325, 350-355.

**Minor comment 5:** *The authors assume 18 years to be the universal adult age, and this is not true even within some LMIC countries. So, it’s better to write it explicitly as 18 years through the manuscript in place of saying “adults”*

**Response:** We thank the reviewers for pointing this out. We have explicitly stated that we mean 18 years or above when referring to adults throughout the revised manuscript. These changes can for example be found on page 5, lines 106-107.
